# P-2169. Integrative scRNA-seq and Transcriptomic Analysis Reveals Monocyte/Macrophage Activation Drives EV-A71-Induced Immune Dysregulation and Neural Injury in Severe HFMD

**DOI:** 10.1093/ofid/ofaf695.2332

**Published:** 2026-01-11

**Authors:** Muqi Wang, Meng Zhang, huiling Deng, Yufeng Zhang, Chenrui Liu, Yuan Chen, Chuting Zhang, Wen Zhang, Xiaoli Jia, Shuangsuo Dang, Yaping Li

**Affiliations:** The Second Affiliated Hospital of Xi'an Jiaotong University, Xi'an, Shaanxi, China; The Second Affiliated Hospital of Xi'an Jiaotong University, Xi'an, Shaanxi, China; Xi'an Children's Hospital, Xi'an, Shaanxi, China; Xi'an Children's Hospital, Xi'an, Shaanxi, China; Xi'an Jiaotong University Second Affiliated Hospital, Xi'an, Shaanxi, China; Xi'an Children's Hospital, Xi'an, Shaanxi, China; Xi'an Jiaotong University Second Affiliated Hospital, Xi'an, Shaanxi, China; Xi'an Jiaotong University Second Affiliated Hospital, Xi'an, Shaanxi, China; Xi'an Jiaotong University Second Affiliated Hospital, Xi'an, Shaanxi, China; Second Hospital of Xi'an Jiaotong University, Xi'an, Shaanxi, China; Xi'an Jiaotong University Second Affiliated Hospital, Xi'an, Shaanxi, China

## Abstract

**Background:**

Enterovirus 71 (EV-A71) is a major pathogen of severe hand, foot and mouth disease (HFMD) in children, but the mechanism by which it develops into severe HFMD remains unclear, especially the role of macrophage-mediated immune dysregulation.

**Figure 1.**
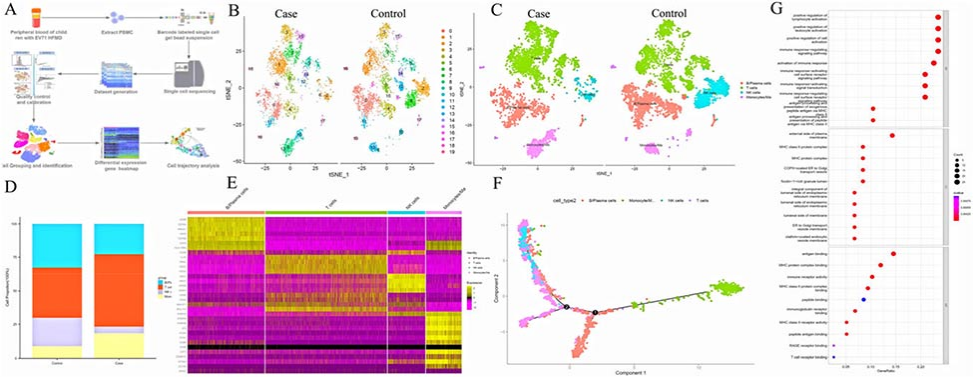
The proportion of peripheral blood mononuclear cells in the HFMD patient at the acute stage compared with that in the healthy control. A: Workflow of single-cell sequencing. B: The distribution of PBMC subsets is shown. C: Different types of immune cells are annotated. D: Proportions of different types of immune cells. E: The marker genes of each cell subpopulation are shown. F: Cell trajectory analysis suggested dynamic changes in and migration trajectories of immune cells during disease progression. G: GO analysis of DEGs with simulated temporal variation. NK cells: natural killer cells; Mø: macrophages.

**Figure 2.**
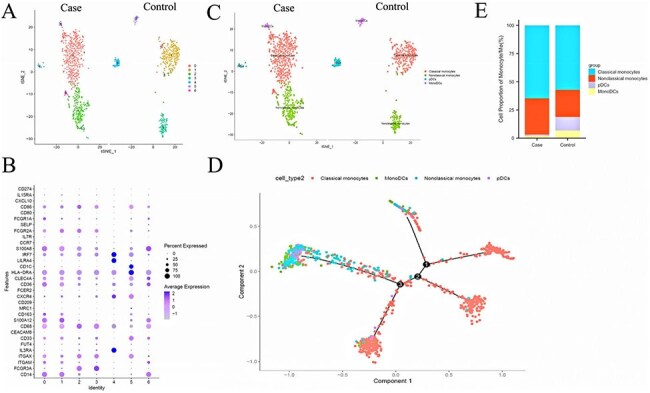
Further monocyte/macrophages cluster annotation. A: Monocyte/macrophages were further classified into 7 subgroups. B-C: Different types of monocyte/macrophages types are annotated. D: Expression of differentially expressed genes in different types of monocyte/macrophages. E: Proportions of different types of monocyte/macrophages. F: Cell trajectory analysis of monocyte/macrophages. G: GO analysis showing the functional annotation of monocyte/macrophages subset. pDCs: plasmacytoid dendritic cells; monoDCs: monocyte-derived dendritic cells.

**Methods:**

Bioinformatics tools were utilized to analyze the transcriptome sequencing results of peripheral blood monocytes (PBMCs) infected with different titers of EV-A71 at various time points. Single-cell sequencing technology was used to sequence obtained PBMCs from a severe HFMD patient due to EV-A71 and a healthy control. Macrophages infected with EV-A71 were collected for transcriptomic analysis, and were indirectly co-cultured with nerve cells to observe their inhibitory effects on nerve cells.

**Figure 3.**
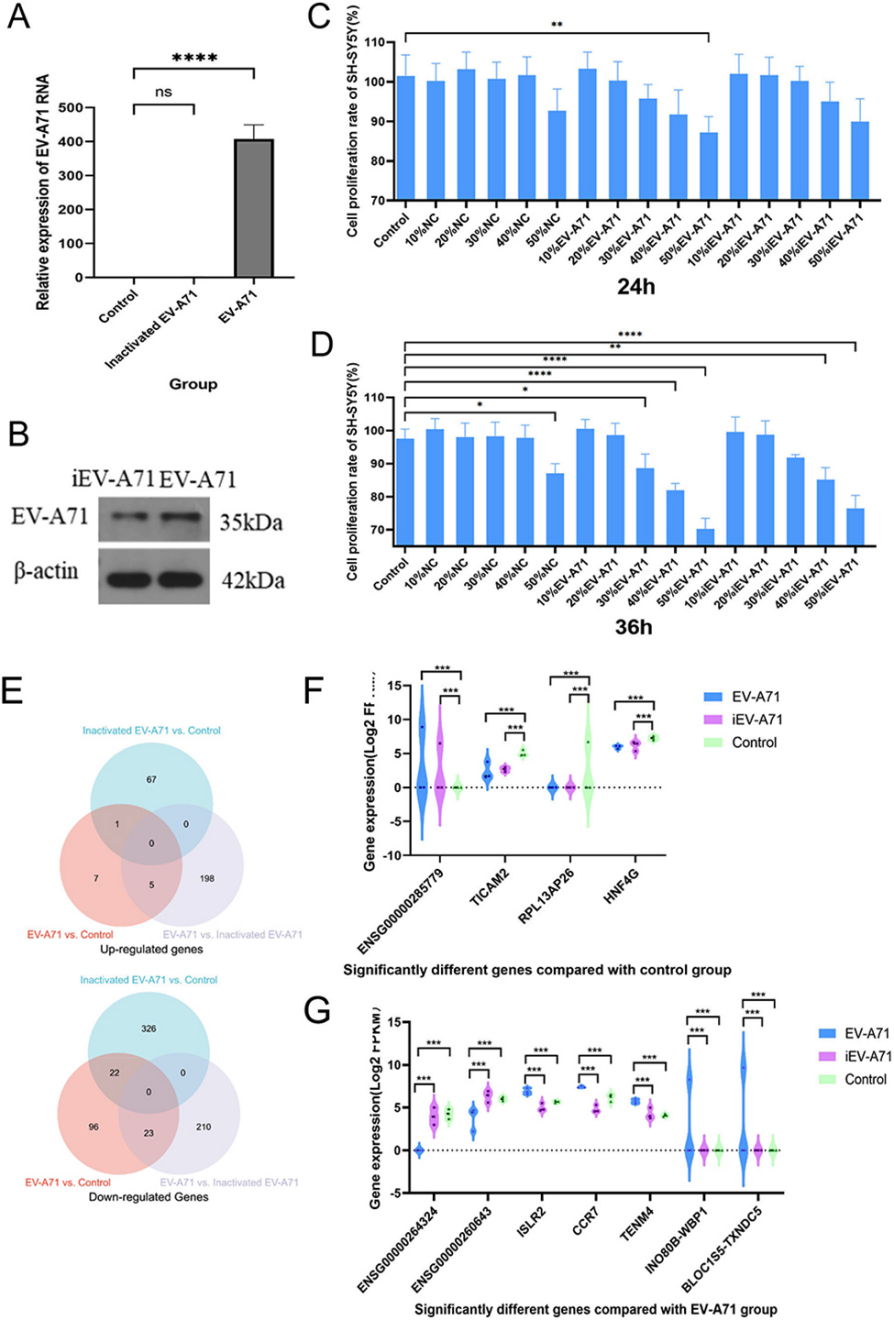
Indirect co-culture of macrophages infected with EV-A71 inhibits the proliferation of nerve cells and differentially expresses genes after infection A: EV-A71 RNA detection. B: EV-A 71 protein detection. C: The cell proliferation rate of the supernatant of EV-A71-infected macrophages co-cultured with SH-SY5Y for 24 hours. D: The cell proliferation rate of the supernatant of EV-A71-infected macrophages co-cultured with SH-SY5Y for 36 hours. NC, normal control, macrophage supernatant co-cultured with SH-SY5Y; iEV-A71, inactivated EV-A71, the supernatant of inactivated EV-A71-infected macrophages co-cultured with SH-SY5Y; EV-A71, the supernatant of EV-A71-infected macrophages co-cultured with SH-SY5Y. E: The number of differentially expressed genes (DEGs) in each group. F: Significantly different genes compared with control group. G: Significantly different genes compared with EV-A71 group.

**Figure 4.**
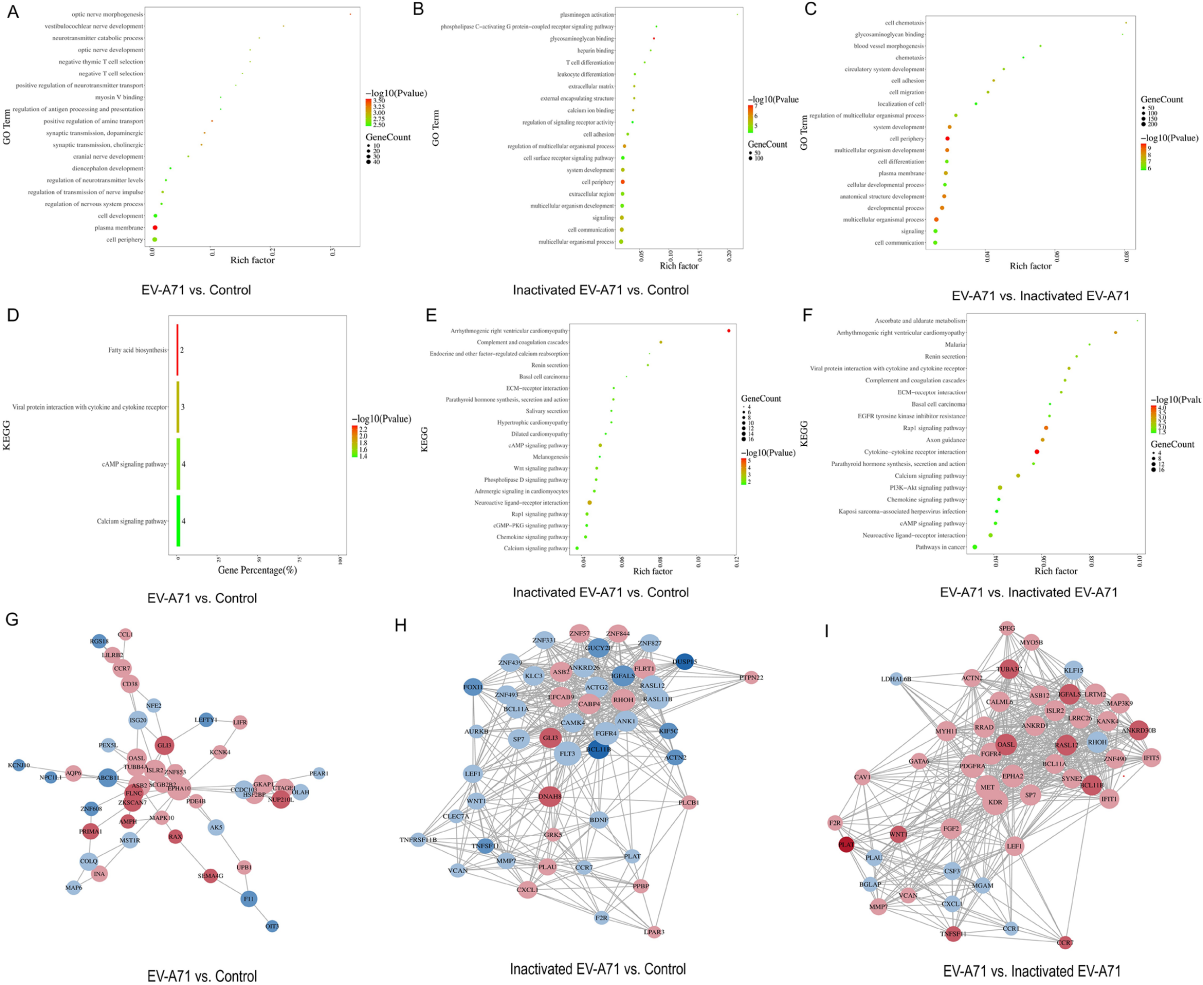
GO analysis, KEGG analysis and protein-protein interaction of differentially expressed genes. A: GO analysis of differentially expressed genes (DEGs) between EV-A71 group and Control group. B: GO analysis of DEGs between Inactivated EV-A71 group and Control group. C: GO analysis of DEGs between Inactivated EV-A71 group and Inactivated EV-A71 group. D: KEGG analysis of DEGs between EV-A71 group and Control group. E: KEGG analysis of DEGs between Inactivated EV-A71 group and Control group. F: KEGG analysis of DEGs between Inactivated EV-A71 group and Inactivated EV-A71 group. G: Protein-protein interaction (PPI) of DEGs between EV-A71 group and Control group. H: PPI of DEGs between Inactivated EV-A71 group and Control group. I: PPI of DEGs between Inactivated EV-A71 group and Inactivated EV-A71 group.

**Results:**

Single-cell RNA sequencing (scRNA-seq) revealed that EV-A71 infected severe HFMD patient had higher monocyte and macrophage ratio (18.50% vs. 8.85%), especially classical (64.59% vs. 57.24%) and non-classical (32.23% vs. 23.90%) monocytes, and a lower pDC (1.19% vs. 12.01%) and monoDC (1.98% vs. 6.80%) in EV-A71 infected severe HFMD patient. Dynamic analysis of PBMCs infected with EV-A71 isolates (mild, moderate and severe) and cell trajectory analysis indicated during infection, monocyte/macrophages were initially activated, followed by three groups of T cells and NK and B cells, M1 macrophage. High concentration of EV-A71 infected macrophage supernatant inhibited SH-SY5Y cell proliferation. ENSG00000285779, TICAM2, RPL13AP26 and HNF4G are significantly different in EV-A71 or inactivated EV-A71 infected macrophages than in control. ENSG00000264324, ENSG00000260643, ISLR2, CCR7, TENM4, INO80B-WBP1, BLOC1S5-TXNDC5 are potential genes about direct virus damage or viral RNA recognition in macrophages. GO annotation and KEGG analysis indicate that EV-A71 infection cause the changes of neural receptor-ligand binding pathway, activation of specific immunity, calcium signaling pathway, and cell aggregation.

**Conclusion:**

Macrophages are activated early during EV-A71 infection, thus initiating specific immunity, which is closely related to the severe HFMD. The nerve damage pathway and calcium signaling pathway caused by EV-A71 virus infection of macrophages deserve to more attention.

**Disclosures:**

All Authors: No reported disclosures

